# The early hunting dog from Dmanisi with comments on the social behaviour in Canidae and hominins

**DOI:** 10.1038/s41598-021-92818-4

**Published:** 2021-07-29

**Authors:** Saverio Bartolini-Lucenti, Joan Madurell-Malapeira, Bienvenido Martínez-Navarro, Paul Palmqvist, David Lordkipanidze, Lorenzo Rook

**Affiliations:** 1 Earth Science Department, Paleo[Fab]Lab, University of Florence, Via G. La Pira 4, 50121 Firenze, Italy; 2 Natural History Museum, University of Florence, Via G. La Pira 4, 50121 Firenze, Italy; 3 Institut Català de Paleontologia Miquel Crusafont, Universitat Autònoma de Barcelona, Edifici ICTA-ICP, Edifici ICTA-ICP, c/ de les columnes s/n Campus de la UAB, Cerdanyola del Vallès, Barcelona 08193 Spain; 4 Department of Geology, Facultat de Ciències, Universitat Autònoma de Barcelona, Edifici C, Campus de la UAB, 08193 Cerdanyola del Vallès, Barcelona Spain; 5 IPHES, Institut Català de Paleoecologia Humana i Evolució Social, C/ Marcel.lí Domingo s/n, Campus Sescelades, Edifici W3, 43007 Tarragona, Spain; 6 Area de Prehistòria, Universitat Rovira i Virgili (URV), Avda. Catalunya 35, 43002 Tarragona, Spain; 7 ICREA, Pg. Lluís Companys 23, 08010 Barcelona, Spain; 8 Departamento de Ecología y Geología, Universidad de Málaga, Campus de Teatinos, 29071 Málaga, Spain; 9 Georgian National Museum, 3, Rustaveli ave., 0105 Tbilisi, Georgia; 10 Tbilisi State University, 1, Tchavtchavadze Avenue, 0179 Tbilisi, Georgia

**Keywords:** Anthropology, Palaeontology, Social evolution

## Abstract

The renowned site of Dmanisi in Georgia, southern Caucasus (ca. 1.8 Ma) yielded the earliest direct evidence of hominin presence out of Africa. In this paper, we report on the first record of a large-sized canid from this site, namely dentognathic remains, referable to a young adult individual that displays hypercarnivorous features (e.g., the reduction of the m1 metaconid and entoconid) that allow us to include these specimens in the hypodigm of the late Early Pleistocene species *Canis* (*Xenocyon*) *lycaonoides*. Much fossil evidence suggests that this species was a cooperative pack-hunter that, unlike other large-sized canids, was capable of social care toward kin and non-kin members of its group. This rather derived hypercarnivorous canid, which has an East Asian origin, shows one of its earliest records at Dmanisi in the Caucasus, at the gates of Europe. Interestingly, its dispersal from Asia to Europe and Africa followed a parallel route to that of hominins, but in the opposite direction. Hominins and hunting dogs, both recorded in Dmanisi at the beginning of their dispersal across the Old World, are the only two Early Pleistocene mammal species with proved altruistic behaviour towards their group members, an issue discussed over more than one century in evolutionary biology.

## Introduction

Wild dogs are medium- to large-sized canids that possess several hypercarnivorous craniodental features and complex social and predatory behaviours (i.e., social hierarchic groups and pack-hunting of large vertebrate prey typically as large as or larger than themselves). Two extant species of wild dogs survive in the Old World, the Indian dhole, *Cuon alpinus* (Pallas, 1811), and the African hunting dog, *Lycaon pictus* (Temminck, 1820). Both are nowadays endangered or critically endangered according to the IUCN red list of threatened species^1,2^. The African hunting dog, known also as painted dog, and the dhole are among the top predators in their respective habitats^3,4^ thanks to the combination of several dental hypercarnivorous traits, skeletal adaptations to cursorial pack hunting and their highly developed social behaviour.

The evolution of these hypercarnivorous canids is still unknown and open to debate^5,6^.

Furthermore, there is also a great deal of confusion in the taxonomy of the extinct large-sized and hypercarnivorous canids, which use to be referred to different systematic denominations (see Supplementary Information). Such names often hint implied or proposed affinities to extant taxa, yet seldomly based on phylogenetic analyses. Considering the results of molecular phylogenies^7,8^, from which it is evident that *Lycaon* and *Cuon* are sister taxa of the crown group of *Canis*, and that the large-sized members of the genus *Xenocyon* might be related to both *Lycaon* and *Cuon*, here we prefer to avoid names suggestive of a closer relationship to any of both genera, privileging the more parsimonious denomination *Canis* (*Xenocyon*) (for an in-depth discussion of the taxonomical issues, see the Supplementary Information).

**Figure 1:**
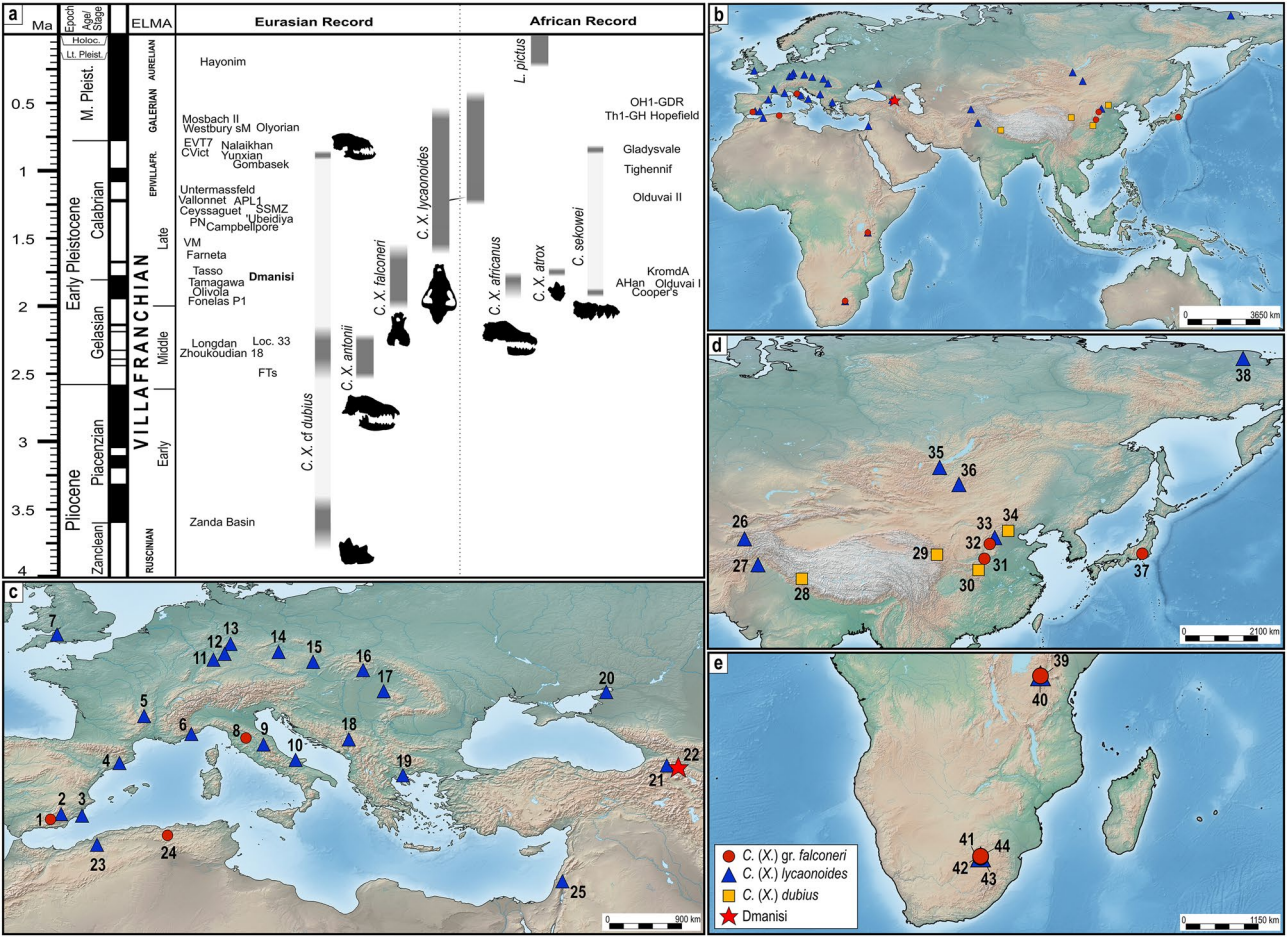
Map and chronology of *Canis* (*Xenocyon*) occurrences. (**a**) Resuming chronological scheme of the known occurrences of fossil wild dogs in the Old World. Abbreviations: AHan, Ain Hanec (Algeria); APL1, Apollonia-1(Greece); CVict, Cueva Victoria (Spain); EVT, Vallparadís Estació (Spain); FTs, Fan Tsun (China); KromdA, Kromdraai A (South Africa); OH1-GR1, Oulad Hamida1-Grotte des Rhinoceros (Morocco); Olduvai I, Olduvai Bed I (Tanzania); Olduvai II, Olduvai Bed II (Tanzania); PN, Pirro Nord (Italy); SSMZ, Shanshenmiaozui (China); Th1-GH, Thomas 1 Quarry-Grotte des Hominides (Morocco); VM, Venta Micena (Spain); Westbury sM, Westbury-sub-Mendip (Great Britain). (**b**,**c**) Maps showing the Old-World occurrences of fossil wild dogs described in the text. (**c**–**e** detailed view of respectively Europe and Circum-Mediterranean area, eastern Asia and southern Africa). Localities: 1, Fonelas-P1 (Spain); 2, Venta Micena (Spain); 3, Cueva Victoria (Spain); 4, Vallparadís Estació (Spain); 5, Ceyssaguet (France); 6, Vallonnet (France); 7, Westbury-sub-Mendip (Great Britain); 8, Upper Valdarno (Italy); 9, Collecurti (Italy); 10, Pirro Nord (Italy); 11, Mosbach II (Germany); 12, Würzburg-Schalksberg (Germany); 13, Untermassfeld (Germany); 14, Koněprusy C178 (Czech Republic); 15, Stránská Skála (Czech Republic); 16, Gombasek (Slovakia); 17, Betfia (Romania); 18, Trlica (Montenegro); 19, Apollonia-1 (Greece); 20, Margaritovo (Russia); 21, Akhalkalaki (Georgia); 22, Dmanisi (Georgia); 23, Tighennif/Terfine (Algeria); 24, Ain Hanec (Algeria); 25, ‘Ubeidiya (Israel); 26, Lakhuti-2 (Tajikistan); 27, Campbellpore (Pakistan); 28, Zanda Basin (China); 29, Longdan (China); 30, Yunxian (China); 31, Loc. 33 in Zdansky (1924) (China); 32, Fan Tsun/Taigu (China); 33, Ma Fang (China); 34, Zhoukoudian 18 (China); 35, Zasukino (Russia); 36, Nalaikha (Mongolia); 37, Tamagawa (Japan); 38, Olyorian fauna (Russia); 39, Olduvai Bed I (Tanzania); 40, Olduvai Bed II (Tanzania); 41, Cooper’s Cave (South Africa); 42, Kromdraai A (South Africa); 43, Gladysvale (South Africa); 44, Hopefield (South Africa). Symbol and colors code (see also graphic legend): red star, Dmanisi site; dark red circles, *C.* (*Xenocyon*) ex gr. *falconeri*; blue triangles, *C.* (*Xenocyon*) *lycaonoides*; yellow squares, *C.* (*Xenocyon*) *dubius*. Chronological scale edited by S. Bartolini-Lucenti in Inkscape ver. 0.92 (https://inkscape.org/) from Bartolini-Lucenti & Madurell-Malapeira^12^. Georeferenced maps (points and background) made in Simplemappr (https://www.simplemappr.net/) and modified in Inkscape ver. 0.92.

The earliest record of a species of this group of hypercarnivorous canids corresponds to *Canis* (*Xenocyon*) cf. *dubius* (Teilhard de Chardin, 1940), which is represented by a single hemimandible^6^ from the Zanda Basin (3.81–3.42 Ma; Fig. 1). The species *C.* (*Xenocyon*) *dubius* is generally related to the lineage of *Cuon*^6,9^. A younger but more complete specimen from Fan Tsun (Taigu^10^) was ascribed to *Canis* (*Xenocyon*) *antonii* (ca. 2.5 Ma)^11^. The latter canid is large-sized and displays evident dental features hinting to an incipient adaptation to a hypercarnivorous diet. Other records of large-sized canids with hypercarnivorous features are rather scanty across Eurasia and are of difficult attribution, considering the presence of hypercarnivorous *Canis* s.s. in Asia during the Early Pleistocene, e.g., *Canis chihliensis* Zdansky, 1924; *Canis teilhardi* Qiu et al., 2004; or *Canis yuanmoensis* You & Qi, 1973.

Around 2.0–1.8 Ma, different forms appeared in several parts of the Old World. These forms showed distinctive dental features (i.e., broad and stoutly-built carnassials with enlarged buccal cuspids), coupled with craniomandibular ones (robust mandibles and developed frontal sinuses). Their large size combined to these dental adaptations could have determined an advantage over the contemporaneous, medium-sized mesocarnivorous canids, as testified by the westward dispersion and radiation of *Canis* (*Xenocyon*) *falconeri* (Forsyth Major, 1877) in Western Europe and of *Canis* (*Xenocyon*) *africanus* (Pohle, 1928) from Olduvai Bed I (Tanzania) or Ain Hanec (Algeria) in Africa. A record of a primitive wild dog attributed to *C.* (*Xenocyon*) cf. *falconeri* was also reported from deposits of Tamagawa (near Tokyo^13^), correlated to 2.1–1.6 Ma^14^. A close relationship between both taxa was suggested by^5,11^ who regarded them as the ancestor of modern *L. pictus.* However, such interpretation has not been shared by other researchers^15^. Recently, a new large-sized taxon was described as *Lycaon sekowei* Hartstone-Rose et al., 2010, based on fragmented cranial material from Cooper’s Cave in South Africa (ca. 1.9 Ma) and an almost complete skeleton from Gladysvale (ca. 1.0 Ma)^16^. Some of the morphologies of the holotype from Cooper’s Cave (i.e., the high-crowned upper premolars, their mesial occlusal morphology, the lingual projection of P4 protocone, and the relative buccolingual length of the M1) cast doubts on its taxonomical attribution and its actual relation with *Canis* (*Xenocyon*)’s group. Moreover, the upper teeth resemble those of the Asian *C. chihliensis*, a large-sized canid possibly belonging to a hypercarnivorous lineage of *Canis*^10^.

During the late Early Pleistocene (i.e., Calabrian stage: 1.8–0.8 Ma), while other more primitive species remained in Africa [e.g., *Canis* (*Xenocyon*) *atrox* Broom in Broom & Schepers, 1946 from Kromdraai A; Fig. 1, although possibly synonym of C. (*Xenocyon*) *africanus*^11^] a more derived form of *Canis* (*Xenocyon*) appeared and became widespread across the whole Old World (Fig. 1). *Canis* (*Xenocyon*) *lycaonoides* (Kretzoi, 1938) was a large-sized canid that resembled *C.* (*Xenocyon*) gr. *falconeri* but with more derived craniodental features (e.g., the P4 protocone tends to attach to the tooth; the M1 metaconule is crest-like; the M1 talon is reduced; the m1 hypoconid is enlarged and tends to be centred in the talonid, which functionally represents a lengthening of the trenchant condition of the trigonid; the entoconid is reduced, being represented by a small crest-like cuspulid; and the m3 is single cusped). Its earliest record appears to be that of Venta Micena (Spain^5^, Fig. 1). In spite of its uncertain chronology, this early occurrence suggests an eastern Asian origin for this hypercarnivorous species. Subsequently, during the late Early Pleistocene and the base of the Middle Pleistocene, from ca 1.6 to 0.7 Ma, *C.* (*Xenocyon*) *lycaonoides* became one of the most common and important members of the carnivoran palaeoguild of Eurasia (Fig. 1). Moreover, *C.* (*Xenocyon*) *lycaonoides* dispersed also in Africa, where it is documented in the northern and eastern part of the continent (e.g., Olduvai Bed II; Fig. 1). Considering the overall cranial morphology and its dental features, which confirm the original interpretation by Kretzoi^17^, Martínez-Navarro & Rook^5^ deemed *C.* (*Xenocyon*) *lycaonoides* as strictly related to extant *L. pictus*. Although some scholars do not favour this interpretation^16,18^, similar conclusions were shared by several other scholars^10,19–21^, who supported also a Eurasian origin for the living African hunting dog.

Among extant Carnivora, *Lycaon pictus* has one of the most complex, structured and unique social behaviours^3,22^. As one of the closest relatives to *L. pictus*, *C.* (*Xenocyon*) *lycaonoides*, the Eurasian hunting dog, might have had comparable complex sociality. Carbone and co-authors^23^ showed that the metabolic energy requirements for large-sized species (> 21.5 kg) force them to predate on prey larger than themselves and thus, in hypercarnivorous Canidae, to hunt cooperatively. As such, this element allows us to figure the social behaviour of extinct hypercarnivorous canids, even with limited direct evidence. Nevertheless, apart from indirect and inferred evidence, direct proof of social behaviour in the Eurasian hunting dog have been reported^24,25^.

Here we report the first occurrence of wild dogs from the Georgian site of Dmanisi (Fig. 1; 1.77–1.76 Ma^26^; see Supplementary Information). This locality preserves an outstanding fossil record, both in terms of abundance, completeness of skeletal remains and preservational status, as testified by the recently described molecular phylogeny based on a fossil rhino tooth^27^. In this paper, we describe the newly discovered remains, identifying them taxonomically and interpreting in the frame of Early Pleistocene diversity of *Canis* (*Xenocyon*). Moreover, the site of Dmanisi has yielded the earliest direct evidence of hominin presence out of Africa in their dispersal throughout Eurasia^28,29^ with also indication of complex sociality among individuals of this population^30,31^. The co-occurrence of two highly social species in the same locality around 1.8 Ma, a time of extreme diversification and expansion of the two clades from their centres of origin^5,6^, raises interest in the role played by social behaviour and by mutually-beneficial cooperation and reciprocity in the geographic expansion of these species. Questions to be explored in this paper.

## Results

### Implications for fossil hunting dogs diversity

**Figure 2:**
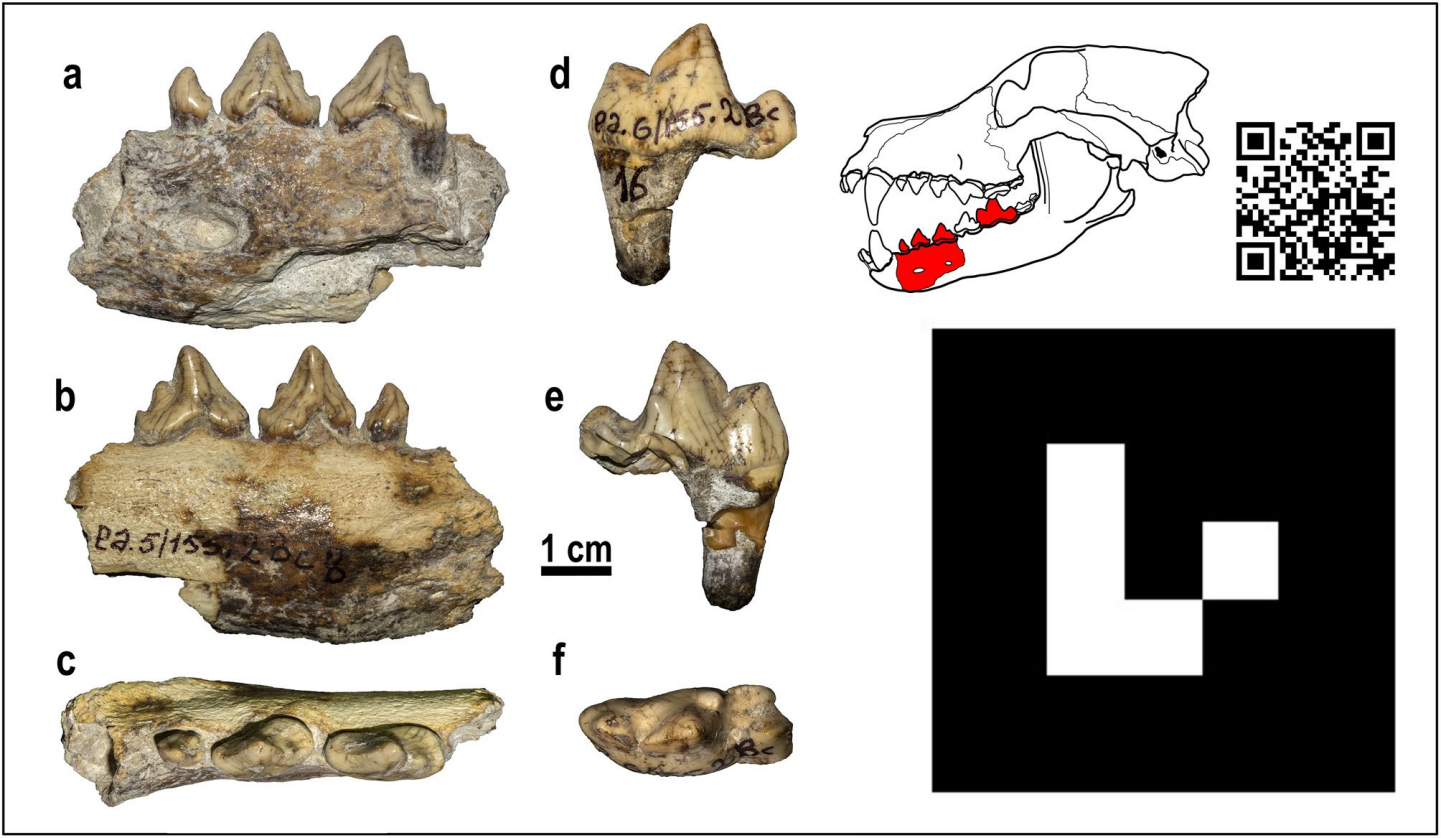
*Canis* (*Xenocyon*) *lycaonoides* from Dmanisi. (**a**–**c**) D6327a, left corpus with p1-p3 in buccal (**a**), lingual (**b**) and occlusal (**c**) views. (**d**)–(**f**), D6327b, left lower m1 in buccal (**d**), lingual (**e**) and occlusal (**f**) views. QR code and Augmented Reality (AR) marker showing 3D comparison between the lower first molar morphologies of *Canis* (*Xenocyon*) from Dmanisi (red), *Canis* (*Xenocyon*) *lycaonoides* from Venta Micena (green) and *Canis* (*Xenocyon*) *falconeri* from Upper Valdarno (gray). Instructions: Scan the QR code on the left; open the link; allow the browser to access the camera of your device; point the camera toward the marker (on the right); and wait for the model to load (up to 10 s). It is possible to turn the device around the marker (or to move the marker) to see different parts of the model. Best visualization performances can be achieved by printing the markers, rather than pointing at them on screens. For common issues refer to Supplementary Information and Bartolini-Lucenti et al.^32^. Photos of the fossil specimens elaborated in Photoshop CC2019 (https://www.adobe.com/). Line drawing of *C.* (*Xenocyon*) and figure composition made by S. Bartolini-Lucenti in Inkscape ver. 0.92 (https://inkscape.org/). AR content made in Visual Studio Code ver. 1.50.0 (https://code.visualstudio.com/) and GitHub Desktop ver. 2.6.6 (https://desktop.github.com/).

The finding of a large-sized canid in the Georgian site of Dmanisi represents an important discovery, which adds valuable information to the current knowledge of canid radiation during the second half of the Early Pleistocene (early Calabrian). Despite the fragmented nature of the specimens, the set of features possessed by D6327 (Fig. 2a–f and Augmented Reality content) allow a confident attribution to *Canis* (*Xenocyon*) *lycaonoides* (see Supplementary Information), the plausible ancestor of the extant African hunting dog^5,19^. As such, this record is the oldest occurrence of Eurasian hunting dogs and precedes the burst of dispersal that the species experienced across the entire Old World during the Calabrian^5,10,19^.

### Dietary preferences of the Dmanisi hunting dog

**Figure 3:**
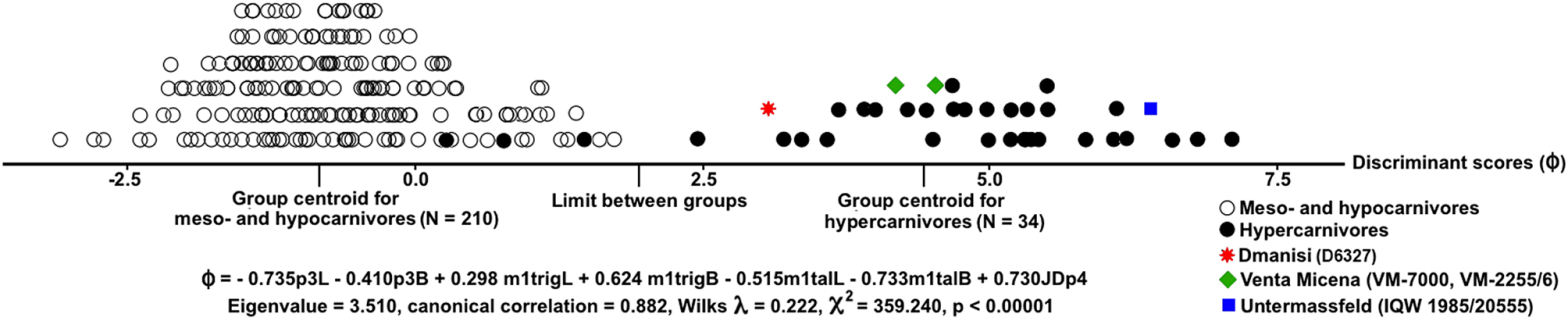
Discriminant analysis using metric measurements of lower teeth (p3 and m1) and jaw between the living omnivorous (i.e., meso- and hypocarnivorous) and hypercarnivorous canids (see metric data available on the online repository at the following link: https://dx.doi.org/dx.10.5281/zenodo.4704327). The scores of the fossil specimens, including Dmanisi, Venta Micena and Untermassfeld, are shown. Graph made in Photoshop CC2019 (https://www.adobe.com/).

In order to test the dietary adaptations of the Dmanisi hunting dog and other Early Pleistocene forms, a linear discriminant analysis was performed over the extant canids (32 species, 247 specimens; craniodental measurements kindly provided by B. Van Valkenburgh), which were grouped in two feeding groups: (i) omnivores (i.e., meso- and hypocarnivores; 27 extant species, 210 specimens), in which vertebrate flesh represents less than 70% of their dietary requirements; and (ii) hypercarnivores (four extant species, 34 specimens), which diet consists almost entirely of vertebrate flesh and are pack-hunters of prey as large as or larger than themselves. Seven metric variables of this dataset for which the measurements were available in the Dmanisi specimens were used in the analysis: length and breadth of the third lower premolar (p3L and p3B, respectively), length and breadth of the trigonid basin of the lower carnassial (m1trigL and m1trigB, respectively), length and breadth of the talonid basin of the lower carnassial (m1talL and m1talB, respectively), and jaw depth measured at the limit between p3 and p4 (JDp4). The linear discriminant function was obtained with the direct method for inclusion of all variables. Reclassification of specimens to each dietary group were derived by cross validations using the leave one out method. After cross-validation, the discriminant function (Fig. 3) correctly allocated 98.8% of the specimens to their feeding group.

Indeed, all omnivores and all hypercarnivores, apart from the four specimens of the small-sized *S. venaticus*, were correctly classified in their feeding groups (Fig. 3). According to the loadings of the variables in the discriminant function, the hypercarnivores show third premolars that are relatively mesiodistally shorter and buccolingually narrower compared to those of omnivorous species, as well as a carnassial with an enlarged trigonid blade and a reduced talonid basin, and a deeper, more stoutly-built mandibular corpus, which is in agreement with previous analyses of adaptations in canids towards hypercarnivory^33,34^. This function reclassified unequivocally the individual from Dmanisi (values of the variables obtained from D6327) in the group of hypercarnivores (Fig. 3), with a probability of pertinence of 0.97. The two specimens of *C.* (*Xenocyon*) *lycaonoides* from Venta Micena (a site that is slightly younger in age than Dmanisi, ca 1.6 Ma) for which these measurements were available were also classified as hypercarnivores. However, they show higher scores in the discriminant function, close to the group centroid of hypercarnivores. Similarly, the single specimen from Untermassfeld, a site of Jaramillo age (ca. 1.0 Ma), shows the highest score among the fossil hunting dogs, which reflects its more advanced adaptations towards hypercarnivory, like those of extant African hunting dogs. These results confirm that the craniodental morphological features of the Eurasian hunting dog from Dmanisi (Fig. 2) were well suited for a diet consisting exclusively of vertebrate flesh. Moreover, they show that there was a gradual evolution of these craniodental adaptations in *C.* (*Xenocyon*) *lycaonoides* from the oldest members analyzed of the lineage (Dmanisi) to the most derived ones (Untermassfeld), confirming the morphological evidence pointed out by other scholars^5,10,19,35^.

## Discussion

Dmanisi, located in the Caucasus at the gates of Europe and near the crossway between Africa and Eurasia, is a key site to explain the dispersal of large mammal species, in a time of great faunal turnovers in the whole Old World^36,37^. This Georgian site also records the earliest direct evidence of hominins presence out of Africa and their dispersal into Eurasia, at ca. 1.8 Ma. Here, we report the record of the Eurasian hunting dog, *C.* (*Xenocyon*) *lycaonoides*, which testifies to the beginning of the dispersal of this more derived, frankly hypercarnivorous canids from its eastern Asia region of origin, similarly to *Canis borjgali* Bartolini-Lucenti et al., 2020 (the mesocarnivorous, wolf-like species also recorded in Dmanisi^32^). During the Calabrian, *C.* (*Xenocyon*) *lycaonoides* became a common element of the entire Old-World faunas in the late Early-early Middle Pleistocene^19^, when it even reached North America^10^. In this dispersal, the Eurasian hunting dog followed at the same time the same dispersal pattern of hominins, just in the opposite direction. The co-occurrences of both species along their dispersal routes together with some other large-sized carnivore taxa, for instance the dirk-toothed cat of African origin *Megantereon whitei* (Broom, 1937)^38,39^, suggest that the ecological conditions favoured the dispersal of these species at that time. Large-sized carnivorans like this felid has been recognized as important supplier of scavengeable resources for the hominins in direct competition with the large-sized scavenger *Pachycrocuta brevirostris*^40,41^.

### Social behaviour of *Canis* (*Xenocyon*) and *Homo* in the late Early Pleistocene

**Figure 4:**
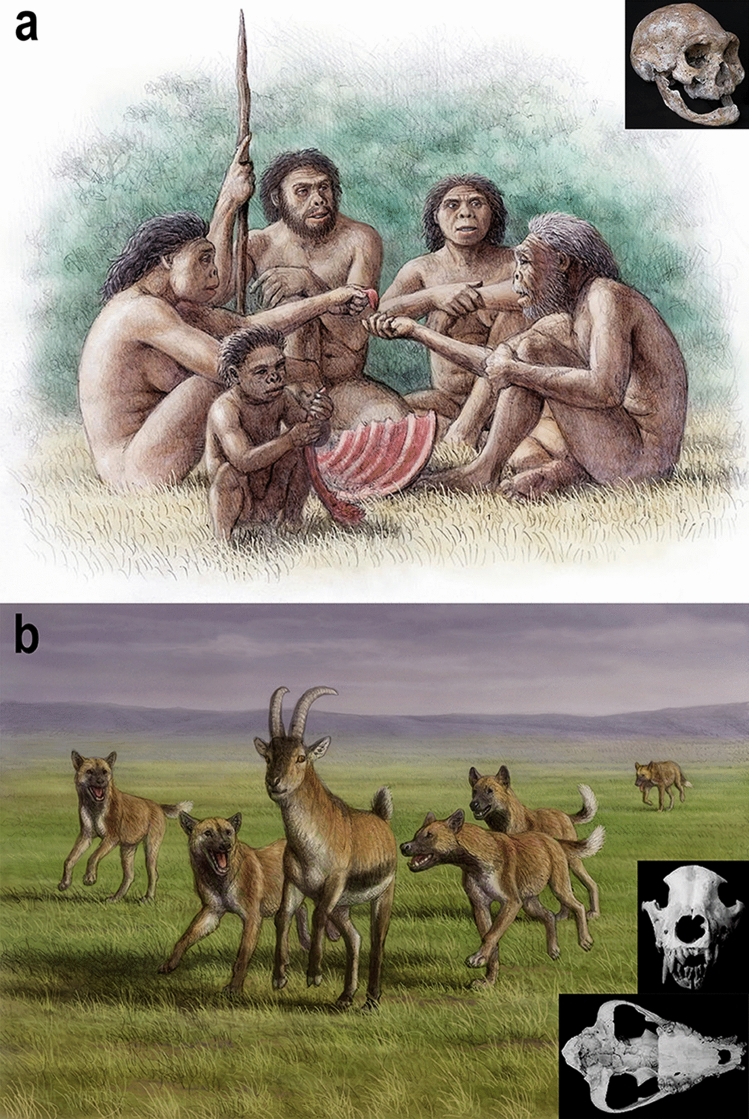
Two social species at Dmanisi. (**a**) altruistic behaviour of a group of *Homo erectus* sharing food with an individual who lived several years without teeth (as evidenced by edentulous skull D3444 and associated mandible D3900). This severe masticatory impairment would limit the diet of the individual to foodstuffs that did not require heavy chewing (e.g., soft plants, animal brain and marrow) or that were orally processed before by others. (**b**) a pack of hunting dogs chasing a prey (goat *Hemitragus albus*) by at Venta Micena, a site where a pathological skull (cranium and associated mandible VM-7000) of *Canis* (*Xenocyon*) *lycaonoides* showing marked bilateral asymmetry and agenesia of several teeth was unearthed. The disabled dog, whose absence of an upper canine probably made it useless for hunting, is drawn running far behind the pack. Given that the individual managed to survive until a relatively advanced age, as indicated by tooth wearing, this suggests that the other members of its family group would have allowed it to feed on the prey captured by the hunting pack. Remains of this hypercarnivorous canid species are also preserved in the assemblage of large mammals from Dmanisi, as shown in this paper. Artwork made by Mauricio Antón with the scientific supervision by the authors of the manuscript.

“There is, at the same time, as much, or perhaps even more, of mutual support, mutual aid, and mutual defense: Sociability is as much a law of nature as mutual struggle”^42^. Probably, the most relevant common feature between the extinct hominins and the fossil hunting dogs is the fossil evidence on the mutually-beneficial cooperation, reciprocity and social behaviour^43^ of both species. This is well documented in Dmanisi by the finding at this site of an edentulous individual of *Homo erectus* (composite skull D 3444/D 3900) who lost all but one of its teeth several years before the time of its death, as evidenced by extensive bone loss in the maxilla and mandible due to resorption of the tooth alveoli. This old individual, probably a female given the relative gracile condition of the skull, could not chew hard or coriaceous food by itself, which means that its survival after the loss of the majority of its teeth probably relied on the assistance from other members of the family group^30^ (Fig. 4a). As it has been noted^30,31^, this kind of altruistic behaviour is beyond forms of biological altruism, proper of non-primate mammals or even “non-human primates”^31^. This suggests that altruistic behaviour and care of the elderly might have developed very early in hominins, at least two million years ago^30,31^. Among Carnivora, social behaviour is frequent, considering the numerous benefits that cooperation offers to carnivorans (increased breeding success and individual survival; enhanced hunting success; ability to kill larger prey; deterrent and strength against kleptoparasites; help for the rearing of pups^44,45^).

Canidae have some of the best-known examples of social organization of all mammals (e.g., the grey wolf, *C. lupus*^46^). Probably less known, yet interesting is the case of the African hunting dogs. This hypercarnivore species display a more complex and peculiar set of behaviour, unique among Canidae, if not carnivorans. This includes exclusive cooperative hunt, obligate cooperative breeding^47^, prioritized access of the pups to the kills^3^, widest variety of vocal repertoire in canids^48^ and consensus decision making via “sneezing”^49^. Many authors^50^ noted a reduced degree of aggressivity between pack members in comparison to other social Canidae (*C. lupus* and *C. alpinus*^46^), even during the consumption of the kill^3^.

Sociality in fossil canids had been investigated by numerous authors^51^ and Carbone and coauthors^23^ proved the necessity for large canids, weighing more than 21.5 kg, to hunt cooperatively to kill on prey larger than themselves. The Eurasian hunting dog *C.* (*Xenocyon*) *lycaonoides* was indeed a large-sized hypercarnivorous species. Body-size estimates suggests that this canid was similar to *L. pictus* (whose average weight is 20–25 kg^52^) if not larger (estimated weight of *C.* (*Xenocyon*) *lycaonoides* = 28 kg^24^). The individual from Dmanisi, despite being a young adult, would have been rather robust (around 30 kg, applying the regression equation for body mass on lower carnassial length^53^). Such a body mass, coupled with its marked hypercarnivorous features, support the idea that *C.* (*Xenocyon*) *lycaonoides* adopted cooperative hunting strategies, similar to the extant canids *C. lupus*, *C. alpinus* and *L. pictus*. Further support of a highly social group organization is provided by fossil pathological specimens. Recently, Tong et al.^25^ described injuries in the sample of Shanshenmiaozui, Nihewan Basin, dated to 1.2 Ma. One of the specimen records a dental infection likely inflicted by processing hard food, such as bone; the other suffered a displaced fracture of its tibia and, despite such a severe injury (which would represent a death sentence for a solitary predator) it managed to survive the trauma to heal. The long period that was presumably required for healing the compound fracture, as well as the incapacitating nature of this trauma for a cursorial predator during the rest of its life (as the healed tibia was considerably shortened), suggests social hunting strategies and provision by other members of the family/pack (primarily food-sharing). Similar pathologies have been also detected in the Late Pleistocene population of *Canis dirus* Leidy, 1858 (recently reassigned to *Aenocyon dirus*^54^) from La Brea tar pits in southern California^55^. This is not surprising considering that packs of extant canids temporarily support wounded or sick members of their group, as reported by many authors in both extant *C. lupus* and *L. pictus*^45^, despite the cost in terms of efficiency of the group^56^. Nevertheless, in the case of African hunting dogs, several studies describe the tolerance by group members not only for injured, but also for disabled or old individuals at the kills^45,57^. Furthermore, disabled or old African hunting dogs receive food by fellow pack members via regurgitation^45,58^, a way of food-sharing that other canids reserve exclusively to kin, very rarely non-kin, pups and to the breeding female. The fossil record yields evidence of similar behaviour in extinct hunting dog as well. An altruistic behaviour of food provisioning to disabled individuals was documented in *C.* (*Xenocyon*) *lycaonoides* at the site of Venta Micena (Fig. 4b). Here a nearly complete cranium with a mandible preserved in anatomical connection were unearthed (skull VM-7000)^24^. This skull belonged to a 7–8 years-old individual (considering the moderate-heavy dental wear of its teeth). By far the most sticking features of this specimen are the high degree of cranial fluctuating asymmetry and several tooth anomalies, including dental agenesia of the upper right canine, the P3 and m3. These teeth were not broken or lost during the life of the individual, as showed by CT scans and radiographs of the cranium^24^. The dental alveolus of the right upper canine is completely absent, as for the other teeth^24^. Moreover, the right m2 is missing and its alveolus is partially reabsorbed. The malformations of the *C.* (*Xenocyon*) *lycaonoides* from Venta Micena were probably due to developmental instabilities resulting from a high level of genetic homozygosity in the relatively small population of wild dogs that inhabited the Baza Basin during late Early Pleistocene times^24^: anodontia (tooth losses) and cranial bilateral asymmetry have been both documented in extant populations of *C. lupus* of small size subject to severe bottlenecks and inbreeding, for example the wolf population of the Białowieża Primeval Forest in Poland^59,60^. In the case of modern *L. pictus,* a study of museum skulls that span a period of a hundred years, which records the dramatic decline in the populations of the species in sub-Saharan Africa during the last century, has shown a marked increase in fluctuating asymmetry as a result of increasing levels of population homozygosity^61^. This suggests that the malformations of the *C.* (*Xenocyon*) *lycaonoides* skull from Venta Micena would reflect developmental instabilities resulting from a high level of genetic homozygosity in the relatively small population of hunting dogs of the Baza Basin, which was geographically (and genetically) isolated from other populations. Moreover, the effective population size of modern painted dogs is typically reduced to 20–35% of the censused population size by reproductive suppression of subordinates and uneven sex ratios^62^. In the case of Venta Micena, this would have also promoted further inbreeding and homozygosity. However, despite the numerous congenital disabilities, the individual VM-7000 was able to reach adulthood, which probably affected or even precluded its ability in the pack-hunting activities (Fig. 4b). This suggests that cooperative behaviour and food provisioning from other members of the family group were the only way for this individual to survive until this age^24^. Similarly to the old human from Dmanisi, who managed to reach such an old age thanks to the altruistic help and care of other family members (Fig. 4a), this hunting dog reached adulthood. This truly altruistic behaviour probably applies also to the hunting dog population of Dmanisi, although the scarce record of this species in the site precludes a direct inference.

Therefore, these findings seem to suggest that increased cooperation and altruistic behaviour may have been important factors for the survival and dispersal of both humans and large social carnivorans in the open environments of Africa, Eurasia and North America. Interestingly, hunting dogs and hominins are up to now the only late Early Pleistocene highly-social species with proved altruistic behaviour towards other members of their group, including food sharing to group members. As noted before, such a behaviour is specially developed in the extant African hunting dog, where individuals with limitations resulting from genetic abnormalities, pathologies and/or advanced age are helped and sustained by the other members of the family group^45,49,50^. *Canis* (*Xenocyon*) *lycaonoides* showed a similar pattern of cooperative and altruistic behaviour towards pack-members^24,25^. The occurrence of the Eurasian hunting dog in Dmanisi marks one the first and better chronologically-constrained record of this large-sized, pack-hunting canid. The success of this wide-ranging dispersion across continents^5,10^, unprecedented and never reached by any other large-sized canids, might be correlated also to the advantages of the mutually-beneficial cooperation and altruistic nature of these extinct hunting dogs, as the result of an evolutionary trend leading to co-operation among members of a species: “the best pathway to advantage for individuals”^63^.

It would not be necessary, but we have here a new evidence of the importance of Dmanisi for that, paraphrasing Dawkins^64^, *Homo* and highly social Canidae both are descended from highly social ancestors and their ancestors lived in groups; this was not an option but an essential survival strategy and from this mutual aid arose.

## Materials and methods

The present study is based on the comparative morphological analysis of the large-sized *Canis* (*Xenocyon*) from Dmanisi and other Plio-Pleistocene hypercarnivorous canids of the Old World. The described fossils are housed at the S. Janashia Museum of Georgia, Georgian National Museum (Tbilisi) (MG-GNM). As comparative fossil material, the Villafranchian and Epivillafranchian canids from the Old World and North America housed at the American Museum of Natural History, New York (United States), Earth Science Dept. of the Aristotle University of Thessaloniki (Thessaloniki, Greece), Institut Català de Paleontologia Miguel Crusafont, Universitat Autonoma de Barcelona (Barcelona, Spain), Museo di Geologia e Paleontologia, Università degli Studi di Firenze (Italy), and Musée National d'Histoire Naturelle (Paris, France) were studied. This fossil comparative sample includes specimens of *Canis* (*Xenocyon*) *dubius* from Zhoukoudian Loc. 18^65^. *Canis* (*Xenocyon*) *falconeri* from Upper Valdarno Basin and Tamagawa^15^. *Canis* (*Xenocyon*) *lycaonoides* from Apollonia-1^66^; Campbellpore^11^; Chukochya^35^, Zanushino^35^; Cripple Creek Sump^10^; Cueva Victoria, Vallparadís Estació^19^; Lakhuti-2^11,35^; Ma Fang^11^; Nalaikha^35^; Olduvai Bed II^5^; Pirro Nord^67^; Shanshenmiaozui^6,25^, Tighennif^68^; Trlica^69^; Untermassfeld^35^; Venta Micena^24^; Westbury-sub-Mendip^70^. *Canis chihliensis* from Yushe Basin^11^. The relevant literature on these canids was reviewed^6,10,13,14,35,57,58,65,72,73^.

Extant specimens housed at the American Museum of Natural History (New York, United States), Museo di Zoologia "La Specola", Università degli Studi di Firenze (Italy), Institut Català de Paleontologia Miguel Crusafont (Barcelona, Spain), Royal Museum for Central Africa (Tervuren, Belgium) and MG-GNM were also used for morphological and metrical comparisons. We examined specimens of *Canis lupus* Linnaeus, 1758, and *Lycaon pictus* (Temminck, 1820). Moreover, a wide data set of craniodental measurements taken in modern canids (247 specimens from 32 species) by Prof. Blaire Van Valkenburgh was used also in some statistical comparisons, including a discriminant analysis between omnivorous (i.e., meso- and hypocarnivorous) and hypercarnivorous canids, in order to deliver palaeoecological inferences for the Dmanisi wild dogs and also for others from different (and younger) sites, like Venta Micena in Spain and Untermassfeld in Germany. Analyses and graphs on dental values present in the supplementary were made in R ver. 3.6.1. (https://cran.r-project.org/) using package ggplot2 ver. 3.2.1 (http://ggplot2.tidyverse.org)^73^.

Cranial and dental measurements were taken with a digital calliper to the nearest 0.1 mm following von den Driesch^74^ with minor modifications.

## Supplementary Information

Supplementary Information.

Supplementary Table S1.

## Data Availability

All data generated or analyzed during the study are included in this published article, in its Supplementary Information Files and on the online repository Zenodo at the following link 10.5281/zenodo.4704327.
