# Supplementary material for: The early hunting dog from Dmanisi with comments on the social behaviour in Canidae and hominins
Source: Sci Rep. 2021 Jul 29;11:13501. doi: 10.1038/s41598-021-92818-4 (PMC8322302; doi:10.1038/s41598-021-92818-4)
Supplement: Supplementary file 1 — Supplementary Information. [file 41598_2021_92818_MOESM1_ESM.docx]

Supplementary Information for

**The early hunting dog from Dmanisi with comments on the social behaviour in Canidae and hominins**

Saverio Bartolini Lucenti^1,2,*^, Joan Madurell-Malapeira^3,4^, Bienvenido Martínez-Navarro^5,6,7*^, Paul Palmqvist^8^, David Lordkipanidze^9,10^, Lorenzo Rook^1^,

*^1^ Earth Science Department, Paleo[Fab]Lab, University of Florence, Via G. La Pira 4, 50121 Firenze, Italy*

*^2^ Natural History Museum, University of Florence, Via G. La Pira 4, 50121 Firenze, Italy*

*^3^ Institut Català de Paleontologia Miquel Crusafont, Universitat Autònoma de Barcelona, Edifici ICTA-ICP, c/ de les columnes s/n Campus de la UAB, Cerdanyola del Vallès, 08193, Barcelona, Spain*

*^4^ Department of Geology, Universitat Autònoma de Barcelona, Facultat de Ciències, Edifici C, Campus de la UAB, 08193 Cerdanyola del Vallès, Barcelona, Spain*

*^5^IPHES, Institut Català de Paleoecologia Humana i Evolució Social, C/ Marcel.lí Domingo s/n, Campus Sescelades, Edifici W3, 43007 Tarragona, Spain*

*^6^ Area de Prehistòria, Universitat Rovira i Virgili (URV), Avda. Catalunya 35, 43002 Tarragona, Spain*

*^7^ ICREA, Pg. Lluís Companys 23, 08010 Barcelona, Spain*

*^8^ Departamento de Ecología y Geología, Universidad de Málaga, Campus de Teatinos, 29071 Málaga, Spain*

*^9^ Georgian National Museum, 3, Rustaveli ave., Tbilisi-0105, Georgia*

*^10^ Tbilisi State University, 1, Tchavtchavadze Avenue, Tbilisi-0179, Georgia*

*joined corresponding authors: Saverio Bartolini Lucenti and Bienvenido Martínez-Navarro

**Email:** [saverio.bartolinilucenti@unifi.it](mailto:saverio.bartolinilucenti@unifi.it); bienvenido.martinez@icrea.cat

1. **Taxonomic remarks on Canis (*Xenocyon*)**

In the last two decades, molecular phylogenies have shown that the hypercarnivorous *L. pictus* and *C. alpinus* are part of a monophyletic clade that includes all the species of the genus *Canis* (i.e., wolves, coyotes, African golden wolves, golden jackals, and Ethiopian wolves^7,8^), whereas the black-backed and the side-striped jackals stem out of this clade and are generally referred to the genus *Lupulella* Hilzheimer, 1906^75^ or to *Lupulella* and *Schaeffia* Hilzheimer, 1906, respectively (if these two African taxa do not belong to a monophyletic clade^76^).

Despite the genetic evidence, the evolutionary history of wild dogs (both African and Asian ones) is still elusive and a matter of debate between scientists, especially due to the fragmentary nature of their fossil record. Indeed, although abundant and widespread across Eurasia, as well as in Africa, from 2.5 Ma until the appearance of the two extant species in the Middle Pleistocene, several alternative taxonomical solutions were used in the last century to accommodate the large-sized hypercarnivorous taxa (as summarized by Wang et al.^6^). For example, the Hungarian paleontologist Milos Kretzoi^17,77^ proposed two extinct genera for the probable ancestors of the extant species: *Xenocyon* Kretzoi, 1938 and *Sinicuon* Kretzoi, 1941. In his view, the former was related to the lineage of the extant African wild dog (as the genotype species is *Xenocyon lycaonoides* Kretzoi 1938) whereas the second would represent the ancestor of *Cuon*, as the name suggests. As Tedford et al.^10^, part of the *querelle* surrounding the taxonomical nomenclature of fossil wild dogs may partially be due to the confusion made by the own Kretzoi when he described both genera. For instance, the name *Sinicuon* *peii* Kretzoi,1941 was used by the Hungarian paleontologist to include the canid material from Zhoukoudian Locality 18 described by Pei^78^. Nevertheless, one year earlier Teilhard de Chardin^65^ had already ascribed the specimens to *Cuon dubius*. Wang et al.^6^ note that the chosen name hints the perplexity that the French paleontologist had to the true relationships between the Zhoukoudian specimens and extant dholes. Perplexity, indeed, reasonably motivated by the evident presence of the m3 on the mandibles from Zhoukoudian Loc. 18. Further complication in this taxonomical and evolutionary history scenario are: the reports by Thenius^79^ of this species (attributed by him to “*Cuon dubius stehlini*”) from the French locality of Rosiéres (later reascribed to *X. lycaonoides* by Schütt, 1973); and the true occurrence of a large-sized dhole (*Cuon alpinus priscus* Thenius, 1954) together with the other hypercarnivorous canid, *X. lycaonoides,* in Mosbach II (Germany^80^). Although the name *Sinicuon* did not catch on much (e.g., used *Cuon*^81^; or used *Xenocyon*^10^), some authors rekindled the interest on this genus. Qiu et al.^9^ attributed a single specimen of a hypercarnivorous canid from Longdan to the new combination *Sinicuon dubius*, following the interpretation of *Sinicuon* as an intermediate form between fossil *Canis* and *Cuon,* as proposed by Kretzoi^77^, whereas *Xenocyon* would relate to *Lycaon*. Sharing this hypothesis, Wang et al.^6^ ascribed the hemimandible fragment from Zanda Basin (Himalayan region) to *Sinicuon* cf. *S. dubius*.

The issue of *Xenocyon* is similarly complicated. When he described the materials from Gombasek, Kretzoi^17^ ascribed the sample of a large-sized canid to two species: “*Canis*” *gigas* Kretzoi, 1938 (:128) and *X. lycaonoides* (Kretzoi, 1938: 132). In 1941, Kretzoi^77^ restudied this fauna and realized the synonymy between the two taxa, favouring *X. gigas* in accordance to the priority rules of zoological nomenclature. The following year, Kretzoi^82^ noticed that the name *Canis gigas* was problematic and therefore chose a new genotypic species name, *Canis spelaeoides* Kretzoi, 1942. Such a name was used only by Musil^83^, in the description of the *Xenocyon* from Stránska Skála, and Schütt^80^ revised the taxonomy of the genus and concluded that *X. lycaonoides* had priority on all other specific names, as the original genotypic species. Schütt’s view is nowadays commonly shared among paleontologists^5,10,11,19,24,35,84^.

Two autoapomorphic features distinguish the extant *L. pictus* and *C. alpinus* from other living Canidae, in addition to derived hypercarnivorous teeth morphologies. The African wild dog does not show the I metacarpal, whereas extant dhole does not retain the m3. Those diagnostic features led several authors to avoid the use of the generic names of extant wild dogs for extinct species with hypercarnivorous adaptations but retaining both of those features, i.e., of uncertain affinity, preferring in their stead *Xenocyon*^10,80^ or *Sinicuon*^6^.

Martínez-Navarro & Rook^5^ proposed to consider the Early Pleistocene *X. falconeri* and *X. lycaonoides* as chronospecies in the phyletic line leading to extant *L. pictus* and, therefore, ascribed them to the genus *Lycaon* (vision shared by^19^). Although considering plausible a strong relationship between these species, especially “*L.*” *lycaonoides*, and *L. pictus*, the inclusion of the extinct species in the genus *Lycaon* renders *Canis* paraphyletic. Similarly, the doubtful interpretation of the large-sized canid remains of Coopers D (1.9-1.6 Ma^85^) and Gladysvale (<700 ka^86^; or ca 1 Ma, unpublished data in^16^) as *Lycaon* *sekowei* Hartstone-Rose et al., 2010, poses taxonomical problems. The latter actually might not even be related to xenocyons for the presence of several morphological traits similar to those of the Asian hypercarnivorous *Canis* *chihliensis* Zdansky, 1924. Some authors^15,87^ chose to include all species of the subtribe Canina in the genus *Canis* (i.e., including black-backed and side-striped jackals), thus reducing the risk of paraphyly. Nevertheless, such an interpretation results unsatisfactory as it does not acknowledge the significant diagnostic morphologies of *Lycaon*, *Cuon* and *Lupulella*. Furthermore, *Xenocyon* may indeed serve as a valid denomination of all intermediate or uncertain Plio-Pleistocene taxa with distinctive hypercarnivorous features. *Sinicuon* could be used in a similar way, but in the original intentions of Kretzoi the specimens of Zhoukoudian Loc. 18 have to be considered closely related to the extant *Cuon*. Therefore, the use of *Sinicuon* has implications for its presumed affinity of fossil specimens/samples^6,9^. Our present knowledge of the true relationships between the extant and extinct species is utterly poor and thus the ascription of fossil forms to a different genus, hardly distinguishable from *Xenocyon* (i.e., there are no current valuable differential diagnosis between *Sinicuon* and *Xenocyon*), seems unsuitable. In order to avoid names suggestive of untested phylogenetic relationship with extant species, and thus privileging more parsimonious denominations, we choose here to follow Rook^11^ and Sotnikova^35^ in considering *Xenocyon* as a subgenus of *Canis.*

1. **Notes on stratigraphic and chronological setting of *Canis* (*Xenocyon*) of Dmanisi.**

Stratigraphic, chronological and taphonomic characterization of the site of Dmanisi has been explained in deep in several works ^26,27,88,89^. The fossil-bearing deposits are covered by medieval ruins and rest on the Mashavera Basalts, which have been dated to 1.85 ± 0.01 Ma using ^40^Ar/^39^Ar dating techniques^26,90,91,92^. The site was historically subdivided into six layers^88^, which were later revised^92^ in two major stratigraphic units, an older A unit and a younger, overlying B unit with further internal subdivisions (numerically designated). Of the numerous trenches and excavations sondages made on the Dmanisi hill top, several works have shown^26,28^ that the stratigraphic scheme of Block 2 and M5 can be regarded as general for the whole Dmanisi site. At least four different ash layers compose the A stratigraphic unit, which conformably overlies the Mashavera basalts (A1-A4 with further internal subdivisions). Given that all A layers show normal geomagnetic polarity, which would correlate to the Olduvai subchron (1.95-1.78 Ma^93^), they can be reliably dated to 1.85-1.78 Ma. The B stratigraphic unit is composed of 5 layers. The first one can be further subdivided into different sublayers, e.g., B1y, B1x and B1z, composed of horizontal ashfalls and pseudokarst pipe and gully fillings^28^ (Fig. S1). All B layers display reversed geomagnetic polarity and correlate, therefore, to the Upper Matuyama chron^26^. The stratified faunal finds in stratum B deposits, including all of the Dmanisi hominins, extend the range of occupations in Dmanisi beyond the Olduvai subchron to ca. 1.77 Ma. Further evidence on the time-constraint of the accumulation of Dmanisi comes from the stratigraphic correlation of the Dmanisi deposits with a thin basalt flow in Zemo Orozmani. This flow is located ca 15 km West of Dmanisi, along the Mashavera^92^ and, more interestingly, it overlies the fossil-bearings sediments of Dmanisi^26,92^. ^40^Ar/^39^Ar dating of this basalt yielded a minimum age of 1.76 Ma ± 0.01 Ma^26,92^. The fossil specimen D6327 comes from the B2 layer (Fig. S1). This layer, as those others from the B stratigraphic unit, can be confidently correlated to a strict timespan of 10 kyr, between 1.77-1.76 Ma^26^.


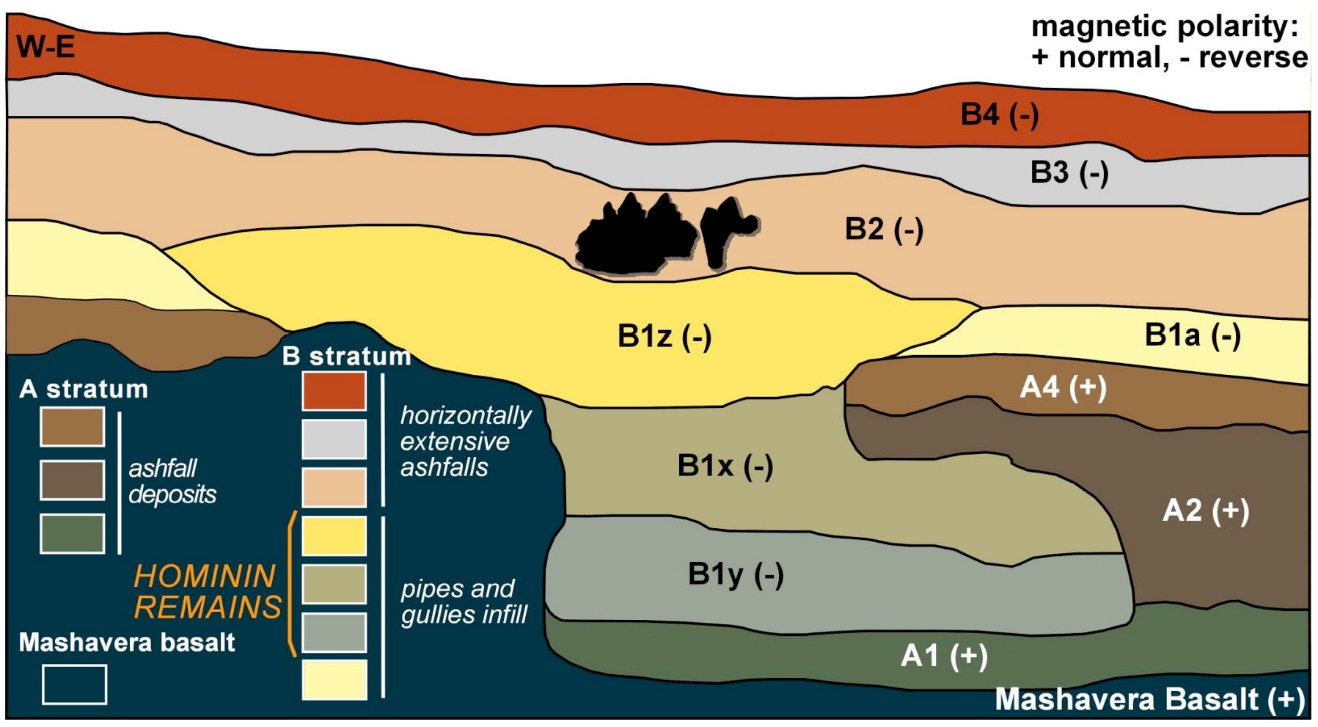


**Figure S1**. Stratigraphic scheme of Block 2 of Dmanisi. Artwork made by S. Bartolini Lucenti after Lordkipanidze et al.^28^ using Photoshop CC2019 (<https://www.adobe.com/>).

1. **Systematic Palaeontology**

Order Carnivora Bowdich, 1821.

Family Canidae Fischer, 1817.

Subfamily Caninae Fisher, 1817.

Tribe Canini Fisher, 1817.

Genus *Canis* Linnaeus, 1758.

Subgenus *Xenocyon* Kretzoi, 1938

*Canis* (*Xenocyon*) *lycaonoides* (Kretzoi, 1938)

Fig. 2, S2-S3

(3D files at https://dx.doi.org/dx.10.5281/zenodo.4704327)

**Referred material—**D6327, left corpus fragment with p1-p3 (D6327a; Fig. S2) and left m1 (D6327b; Fig. S3).

**
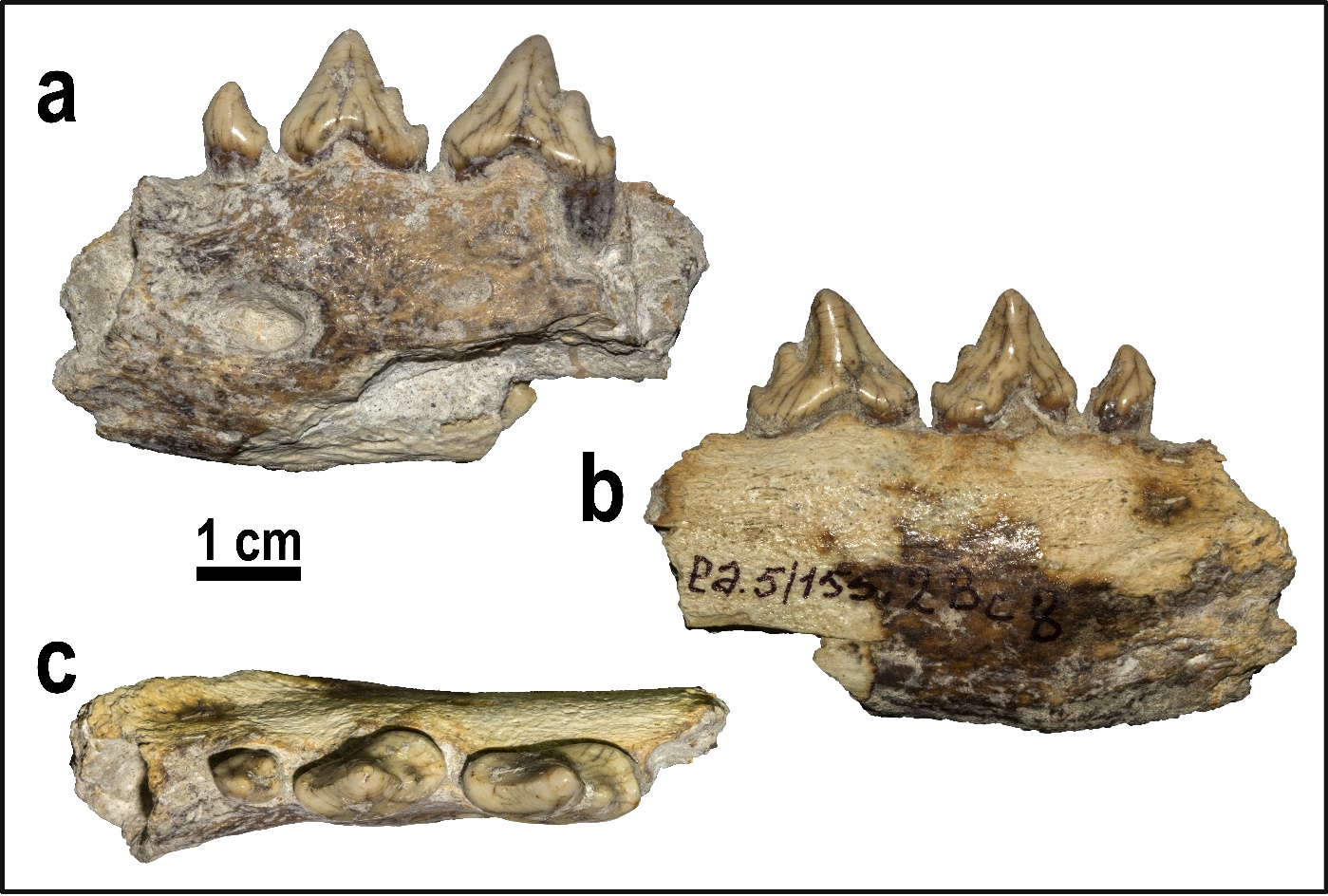
**

**Figure S2.** Detailed figures of the rostral portion of the left hemimandible D6327 (D6327a) in buccal (a), lingual (b) and occlusal (c) views. Photos by S. Bartolini Lucenti, elaborated in Photoshop CC2019 (<https://www.adobe.com/>). The final editing and styling of the figure were made in Inkscape ver. 0.92 (<https://inkscape.org/>).

**
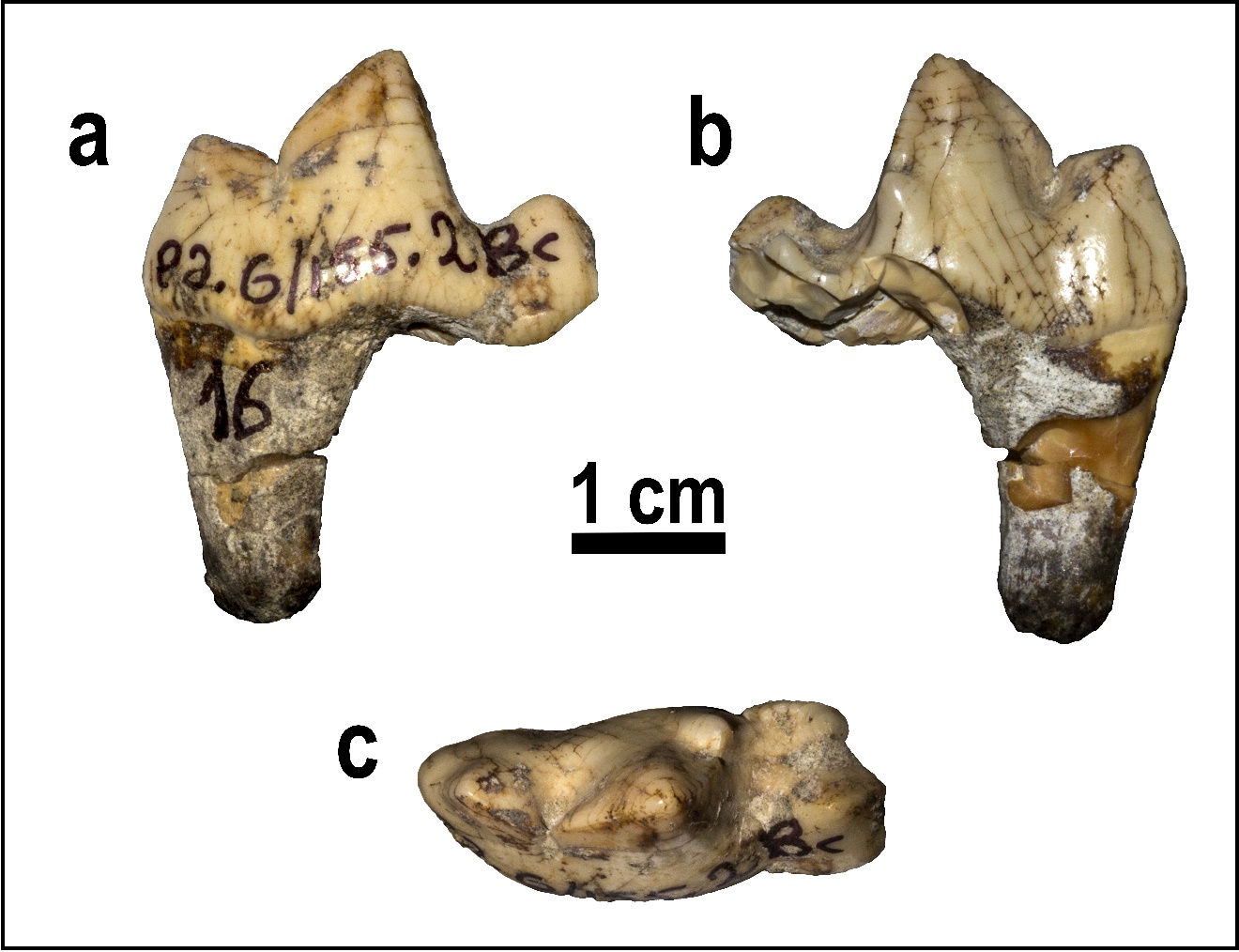
**

**Figure S3.** Detailed pictures of the m1 of the left hemimandible D6327 (D6327b), shown in Fig. 2. The fossil specimen is shown in buccal (a), lingual (b) and occlusal (c) views. Photos by S. Bartolini Lucenti, elaborated in Photoshop CC2019 (<https://www.adobe.com/>). The final editing and styling of the figure were made in Inkscape ver. 0.92 (<https://inkscape.org/>).

**Measurements—**Table S1.

| **Cat. Numb.** | **D6327** | | |
| --- | --- | --- | --- |
|  | L | W | H |
| **p1** | 6.4 | 4.7 | - |
| **p2** | 13.8 | 7.2 | - |
| **p3** | 16.2 | 7.2 | - |
| **m1** | 30.5 | 11.6 | - |
| **trm1** | 20.6 | 11.6 | - |
| **tdm1** | 9.9 | 11.2 | - |
| **Mp1H** | - | - | 28.7 |
| **Mp2H** | - | - | 27.4 |

**Table S1.** Dental measurements of the *Canis (Xenocyon) lycaonoides* specimens from Dmanisi. Abbreviations: H, height; L, mesiodistal length; Mp1H, height of the mandibular corpus at level of the distal side of the p1; Mp2H, height of the mandibular corpus at level of the distal side of the p2td, talonid; tr, trigonid; W, buccolingual width.

**Anatomical Description—** D6327 does not show significant degree of dental wear, which suggests that it belonged to a single individual, probably a young adult (Figs. 2, S2-S3). The corpus fragment of D6327 is rather high and stout. On the buccal side, two foramina are evident. The mesial one is wide and deep, with a distal margin ending under the p2, whereas the distal one, much smaller, ends under the mesial part of p3. There are no diastemata between the premolars. The protoconids of the premolars are high and sharp. The p1 has a single cuspid and an enlarged distolingual portion (Figs. 2, S2). The p2 possesses a small but evident distal cuspulid and a developed distal cingulid. The distal portion of the tooth is consistently enlarged compared to the mesial portion. The p3 is larger than the p2, with a more robust mesial portion (Figs. 2, S2). There is an evident distal accessory cuspulid and a prominent cingulid in the p2. The m1 is stout, in occlusal view, with a large paraconid and a high and sharp protoconid (Figs. 2, S3). The metaconid is closely attached to the protoconid and poorly individualized. In the talonid basin, the hypoconid is almost centralized and the entoconid is present, though considerably reduced, almost to a crest.

**Comparison with other large-sized hypercarnivorous canids of Eurasia—**The studied m1 from Dmanisi shows hypercarnivorous adaptations like the reduction of the metaconid and entoconid. The mandibular fragment is stout and high, like in other Early Pleistocene large-sized canids as *C.* (*Xenocyon*) *antonii* from Fan Tsun^11^, “*C. chihliensis*” from Shanshenmiaozui^25^, *C.* (*Xenocyon*) *falconeri* from Upper Valdarno (Del Campana, 1913) and *C.* (*Xenocyon*) *lycaonoides* from e.g., Venta Micena, Untermassfeld, Vallparadís Estació^19,35,94^ or the extant *C. lupus*, *L. pictus* or *C. alpinus*. Compared to their mesiodistal length, the premolars are higher than in other *Canis* (*Xenocyon*) species, in which the cuspids tend to be shorter. Nevertheless, in some specimens [e.g., VM-2255 and VM-2257, *C.* (*Xenocyon*) *lycaonoides* from Venta Micena^19^], the premolars show a similar morphology. However, this feature does not seem reliable, as it varies considerably in extant *L. pictus* and *C. alpinus*^19^. For instance, in *L. pictus*, 56% of the examined specimens (16 out of 29 specimens) possess more robust and shorter protoconids whereas 47% show thinner and higher ones. The presence of distal accessory cuspulids on the p2 and the p3 is fairly common in Early Pleistocene *C.* (*Xenocyon*) *falconeri* and *C.* (*Xenocyon*) *lycaonoides,* and also in *L. pictus*, whereas it seems uncommon in the Middle Pleistocene forms of *C.* (*Xenocyon*) *lycaonoides*, but also in *C.* (*Xenocyon*) *dubius* and in *C. alpinus*. In contrast to extant *L. pictus*^19^ *C.* (*Xenocyon*) from Dmanisi, *C.* (*Xenocyon*) *falconeri* from Upper Valdarno and *C.* (*Xenocyon*) *lycaonoides* from Eurasia do not possess accessory mesial cuspulids on the p2 or p3, just a sharp cristid on the mesial side of the protoconid.

The m1 is stoutly built, as in *C.* (*Xenocyon*) *falconeri* and *C.* (*Xenocyon*) *lycaonoides,* and larger buccolingually compared to that of *C.* (*Xenocyon*) *dubius* from Zanda Basin^6^, Linyi^81^ and Zhoukoudian Loc. 18^65^. One of the key features of the lower carnassial D6327b is the morphology of the entoconid. In the Dmanisi specimen, in contrast to *C.* (*Xenocyon*) *falconeri*, the entoconid is reduced, but not as reduced as in *C.* (*Xenocyon*) *dubius* from Zanda Basin and Loc. 18, or in some Middle Pleistocene forms of *C.* (*Xenocyon*) *lycaonoides* (e.g., Cripple Sump Creek and Olyorian Fauna^10,72^); Gombasek, Koněprusy C178^17^; Westbury-sub-Mendip^70^; Zanushino and Chukochya^35^. The development of the entoconid of the specimen from Dmanisi is more consistent with earlier forms of *C.* (*Xenocyon*) *lycaonoides*, like those of Early Pleistocene sites as Cueva Victoria, Trlica^69^, Untermassfeld^35^, Vallparadís Estació and Venta Micena^19^ (see AR comparison in Fig. 2; see also Fig. S4).


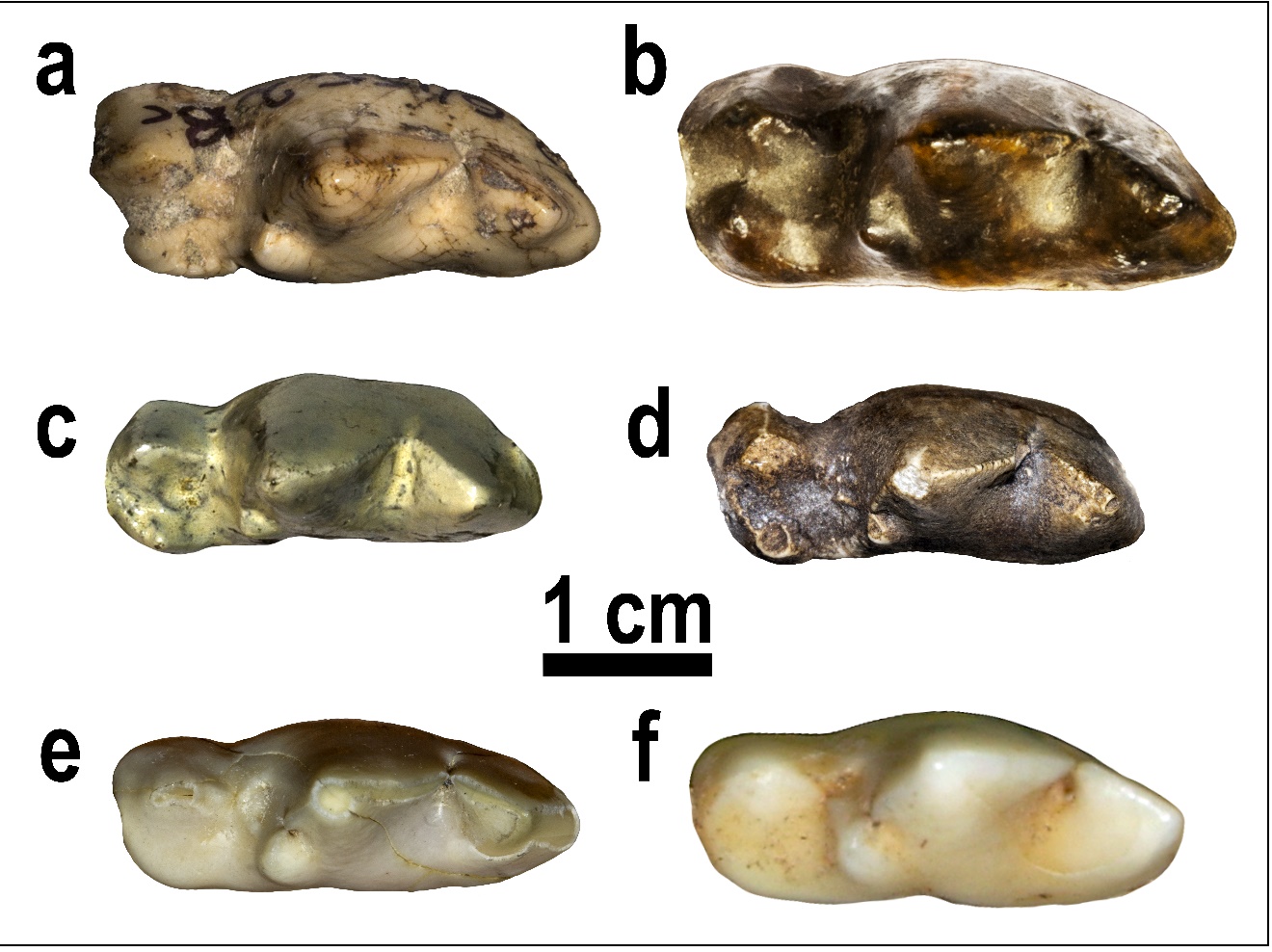


**Figure S4.** Figure comparing the occlusal morphology of the lower carnassial of *C.* (*Xenocyon*) spp. and *Lycaon pictus*. a, D6327, left m1 of *C.* (*Xenocyon*) *lycaonoides* from Dmanisi; b, IGF 865, left m1 of *C.* (*Xenocyon*) *falconeri* from Upper Valdarno; c, VM-2255 left m1 of *C.* (*Xenocyon*) *lycaonoides* from Venta Micena; d, VM-2256 right m1 of *C.* (*Xenocyon*) *lycaonoides* from Venta Micena (reversed); e, AMNH-82082 right m1 of *L. pictus* from Africa (reversed); f RMCA 13143 left m1 of *L. pictus* from Africa. Photos by S. Bartolini Lucenti, elaborated in Photoshop CC2019 (<https://www.adobe.com/>). The final editing and styling of the figure were made in Inkscape ver. 0.92 (<https://inkscape.org/>). Photos by S. Bartolini Lucenti and J. Madurell-Malapeira, elaborated in Photoshop CC2019 (<https://www.adobe.com/>). The final editing and styling of the figure were made in Inkscape ver. 0.92 (<https://inkscape.org/>).

Moreover, as Sotnikova^35^ noted, the reduction of the m1 entoconid in Early Pleistocene *C.* (*Xenocyon*) *lycaonoides*, although remaining an important feature, has a certain degree of variation: in specimens coming from the same sample (e.g., in Lakhuti-2, Ma Fang, Nalaikha, and Untermassfeld) this cuspulid is slightly reduced (e.g., F:AM 97046 from Ma Fang), while in others it is vestigial, only slightly emerging from a lingual cingulid (e.g., F:AM 97045 from Ma Fang). The latter condition is shared, and furtherly reduced, in several specimens from Middle Pleistocene localities, like those mentioned above (i.e., Koněprusy C178, Cripple Sump Creek). The position of the hypoconid in the m1 talonid relates to the development/reduction of the entoconid. In *C.* (*Xenocyon*) *falconeri* and *C.* (*Xenocyon*) *antonii* from Fan Tsun the talonid basin is bicuspidate, with the hypoconid not centred in this portion. Such shared morphology in these two taxa allowed Rook^11^ and Martínez-Navarro & Rook^5^ to suggest a strong relationship between these species [*C.* (*Xenocyon*) gr. *falconeri* in^11^]. In contrast, the hypoconid in *C.* (*Xenocyon*) *lycaonoides* tends to become larger, occupying the majority of the talonid, especially in those specimens where the entoconid is reduced to a lingual cuspulid-like cristid. The m1 talonid basin of the Dmanisi sample shows an almost centralized and considerably reduced hypoconid, a crest-like entoconid, a condition that fits the early *C. (Xenocyon) lycaonoides*. This derived condition results in the transformation of the talonid basin of the lower carnassial from a basin-like depression into a trenchant heel, which represents a functional lengthening of the trigonid blade of the carnassial^28^. In three out of the four living hypercarnivorous canids, the dhole, the painted dog and the South American bush dog (*Speothos venaticus*), the hypoconid is a single, large, centrally positioned, bladelike cusp. In the fourth hypercarnivorous species, the Eurasian and North American grey wolf (*Canis lupus*), the hypoconid is enlarged at the expense of the entoconid, as in *C.* (*Xenocyon*) *lycaonoides,* which represents a less advanced grade in the evolution towards hypercarnivory of a trenchant-like talonid.

Linear measurements of the specimens from Dmanisi show a large size comparable to early taxa and the Early Pleistocene *C.* (*Xenocyon*) *lycaonoides*. An extensive sample of fossil specimens was used to compare the relative length of the m1 trigonid as opposed to the m1 width in the known fossil *Canis* (*Xenocyon*) and living African hunting dogs (Fig. S5).

**
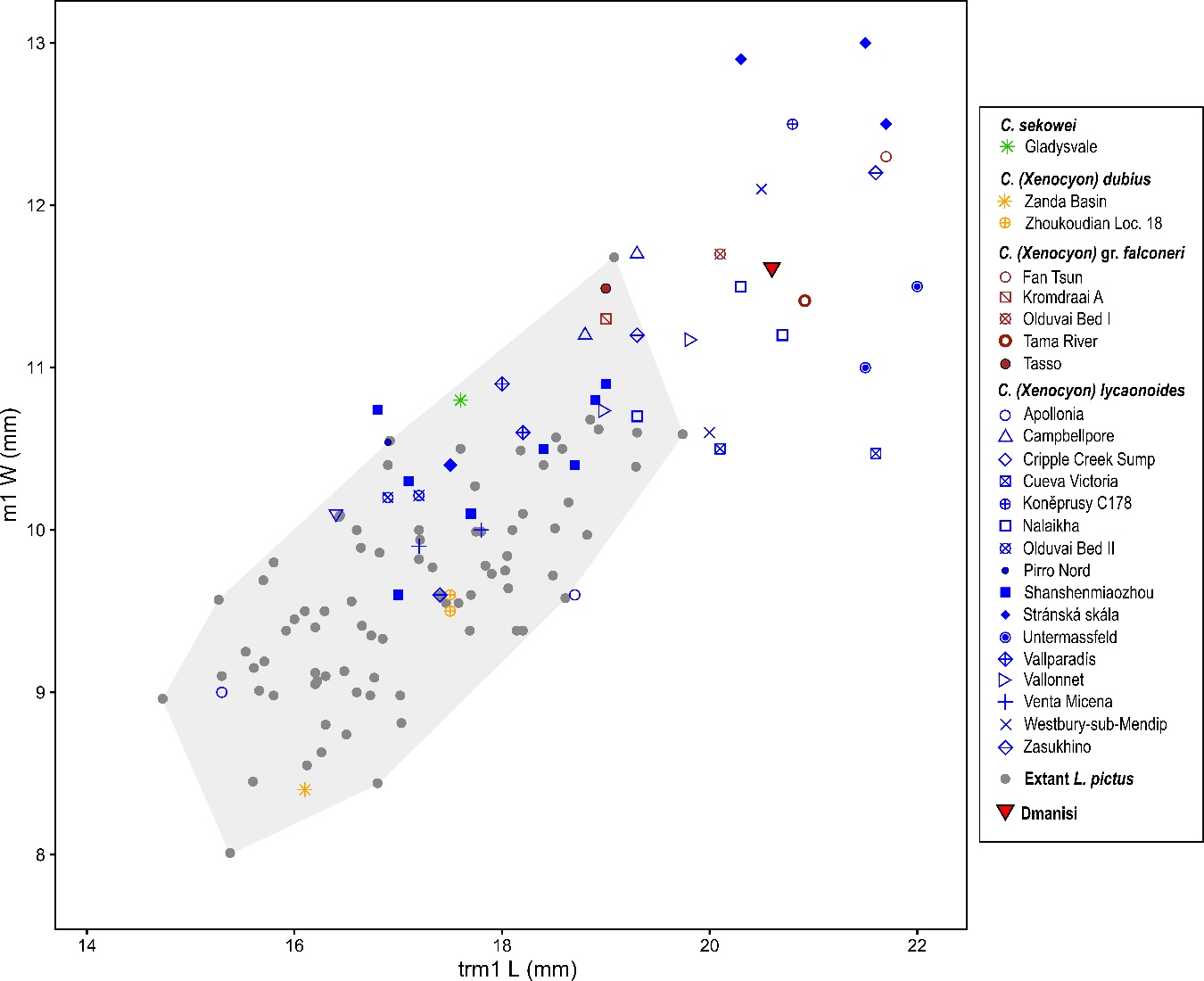
**

**Figure S5.** Scatter-plot comparing m1 trigonid length and m1 width in Pliocene-Early Pleistocene wild dogs and *L. pictus*. Gray shaded area: range of variability for extant African wild dog. Graph obtained using package ggplot2 ver. 3.2.1 (<http://ggplot2.tidyverse.org>)^73^ in R ver. 3.6.1 (https://cran.r-project.org/).

The size variability of the fossil derived *C.* (*Xenocyon*) *lycaonoides* is fairly broad. Moreover, apparently the variability is not only between samples (chronologically referred to a time span of more or less one million years) but also within specimens of the same sample (e.g., Apollonia-1^66^; or Zasukhino^35^). It is worth noting that the variance showed by *C.* (*Xenocyon*) *lycaonoides* is more or less comparable to that of the extant *L. pictus*. This is also confirmed by Fig. S6: the relative size of the lower carnassial of the fossil taxon is of the same degree of magnitude than in *L. pictus*. Unfortunately, earlier forms are poorly represented and known from few specimens, often incomplete, which prevents us from drawing inferences on their variability. Only *C.* (*Xenocyon*) *dubius* possibly increased the stoutness of the m1 in time, yet the low number of specimens discourage any hypotheses in this sense. Dmanisi m1 falls close to *C.* (*Xenocyon*) *africanus* from Olduvai bed I and also to medium- to large-sized *C.* (*Xenocyon*) *lycaonoides*, to which is close for the length of the trigonid.


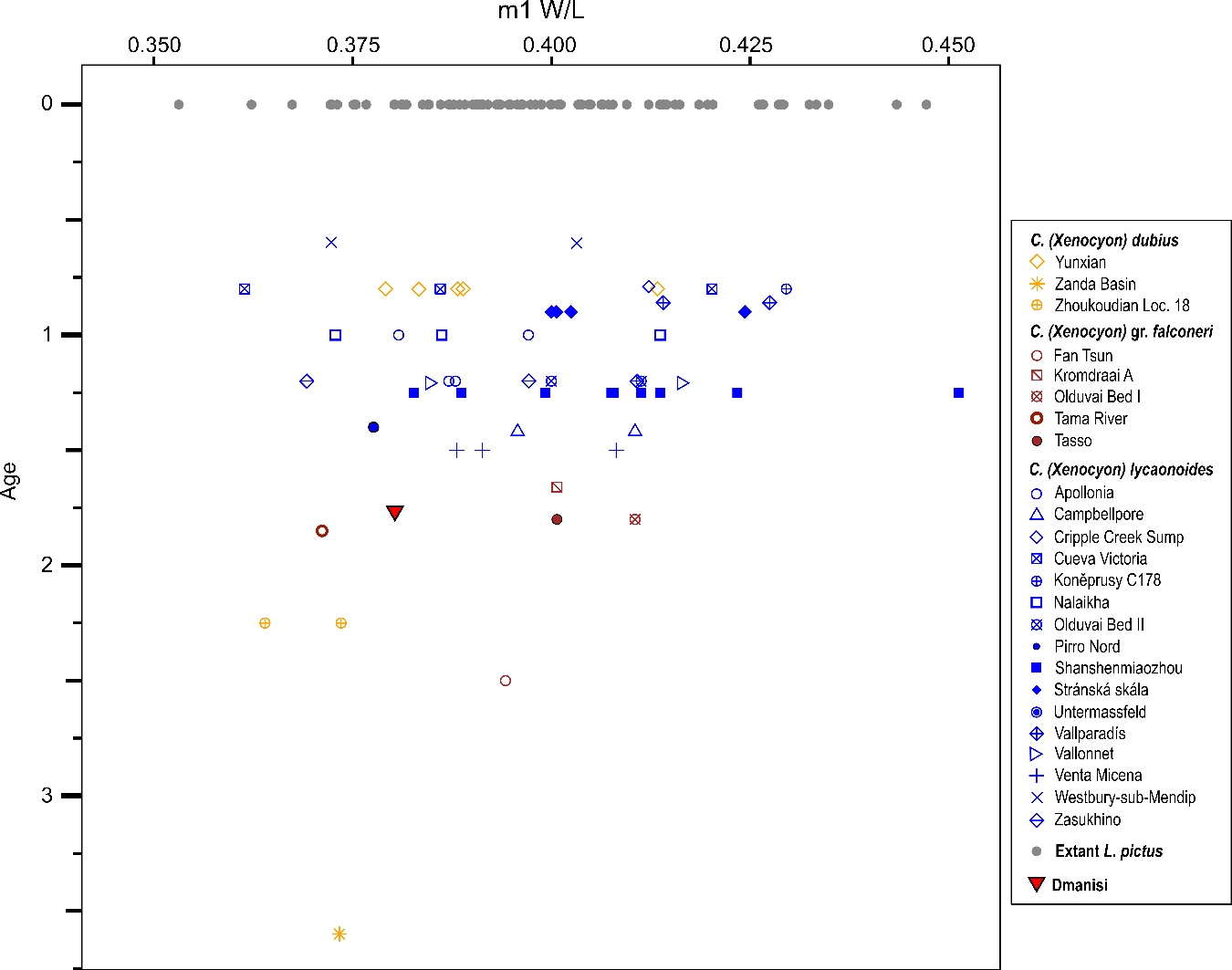


**Figure S6.** Distribution of m1 width and length ratio in fossil wild dogs (of the last 3.75 Ma) and in extant *L. pictus*. Graph obtained using package ggplot2 ver. 3.2.1 (<http://ggplot2.tidyverse.org>)^73^ in R ver. 3.6.1 (https://cran.r-project.org/)

1. **Visualization and trouble-shooting to AR the content of Fig. 2 (following Bartolini Lucenti et al.^32^)**

Example of the visualization of the Augmented Reality web app of the comparison between different *Canis* (*Xenocyon*) is visible in Figure S7.


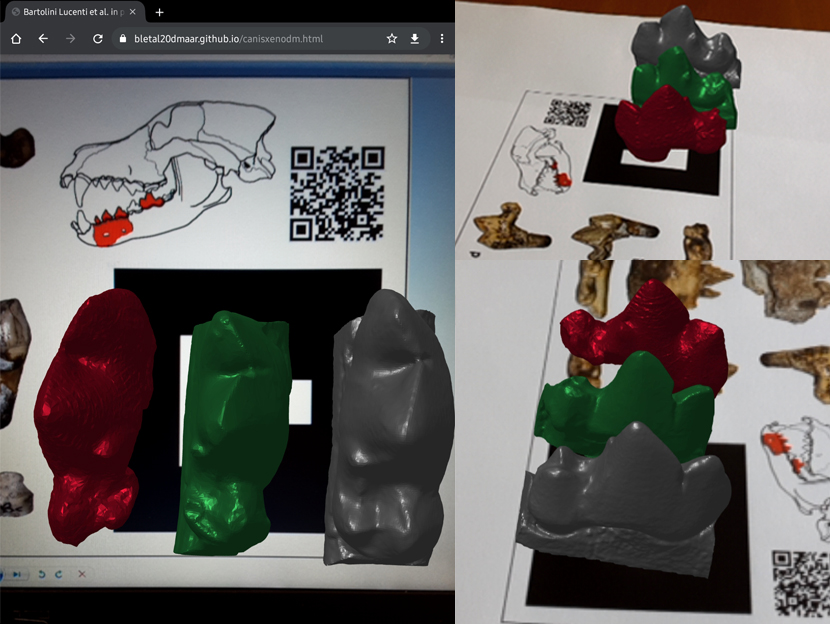


**Figure S7.** AR comparison of lower carnassials of *C.* (*Xenocyon*), using an Android tablet (pointing at the marker of Figure 2). On the left, screenshot of the visualization of 3D teeth on the marker shown a pc screen. On the right, same element but on a printed marker. Photos by S. Bartolini Lucenti, elaborated in Photoshop CC2019 (<https://www.adobe.com/>).

**Guide to use of the AR Web-app**

To visualize the QR-code: choose any free application from the App Store/Play Store. Best performance of the AR visualization can be achieved with printed markers, rather than on screens (pc monitors, tablets, etc.). Prints reducing back-scattered light on the markers represent optimal conditions for the AR visualization.

**iOS devices**

To visualize the Augmented Reality web-app:

1) Allow Safari to open the camera of the iPhone: Go to Setting > Safari > turn on “Camera & Microphone Access” under the “Privacy & Security” submenu.

2) Scan the QR-Code.

3) Open the link in Safari (n.b.: the web-app does not work in Chrome for iOS or other browsers)

4) Confirm the use of the camera by the browser.

5) Point at the marker, wait the for the model to load.

Best model-rendering performances if the scene is in landscape mode (i.e., allowing the phone to automatically rotate the view). N.b. if the model does not appear in landscape mode, although it was visible in portrait mode, refresh the page.

**Android devices**

To visualize the QR-code: choose any free application from the App Store.

To visualize the Augmented Reality web-app:

1) Scan the QR-Code.

2) Open the link in your mobile browser (note that Chrome is preferable)

3) Confirm the use of the camera by the browser.

4) Point at the marker, wait the for the model to load.

Best model-rendering performances if the scene is in landscape mode (i.e., allowing the phone to automatically rotate the view). N.b. if the model does not appear in landscape mode, although it was visible in portrait mode, refresh the page.

**SI References**

1. Lindblad-Toh, K. *et al.* Genome sequence, comparative analysis and haplotype structure of the domestic dog. *Nature* **438**, 803–819 (2005).
2. Zrzavý, J. & Řičánková, V. Phylogeny of recent Canidae (Mammalia, Carnivora): relative reliability and utility of morphological and molecular datasets. *Zool. Scr.* **33**, 311–333 (2004).
3. Kretzoi, M. Weitere Beiträge zur Kenntnis der Fauna von Gombaszög. Ann. Hist. Nat. Mus. Hung. **34**, 105–139 (1941).
4. Pei, W. C. The upper cave fauna of Choukoutien. *Palaeont. Sinica* **10,** 1–84 (1940).
5. Thenius, E. Zur Abstammung der Rotwölfe (Gattung Cuon Hodgson). *Österr. Zool*. **5**, 378–387 (1954).
6. Schutt, G. Revision der *Cuon*-und *Xenocyon*-funde (Canidae, Mammalia) aus den Alt Pleistozänen Mosbacher Sanden (Wiesbaden, Hessen). *Mainzer Naturwissenschaftliches Archiv*. **12**, 49–77 (1973).
7. Wang, S. & Zhao, S. Additional note on the *Cuon* from Linyi, Shanxi province, China In *Proceedings of the Tenth Annual Meeting of the Chinese Society of Vertebrate Paleontology* (Ed. W. Dong) 63–68 (Beijing, China Ocean Press, 2006).
8. Kretzoi, M. Präokkupierte und durch ältere zu ersetzende Säugetiernamen. *Földtany Közl- öny* **72**, 345–349 (1942).
9. Musil, R. Die Caniden der Stránská Skála. *Anthropos* **20**, 77–106 (1972).
10. Sotnikova, M. V. & Vislobokova, I. A. Pleistocene mammals from Lakhuti, Southern Tajikistan, USSR. *Quatärpaläontologie* **8**, 237–244 (1990).
11. Berger, L. R., De Ruiter, D. J., Steininger, C. M. & Hancox, J. Preliminary results of excavations at the newly investigated Coopers D deposit, Gauteng, South Africa: preliminary research reports: human origins research in South Africa. *S. Afr. J. Sci.* **99**, 276–278 (2003).
12. Lacruz, R. S. *et al.,* Palaeontology and geological context of a Middle Pleistocene faunal assemblage from the Gladysvale Cave, South Africa. *Palaeontol. Africana* **114**, 99–114 (2002).
13. Werdelin, L. & Peigné, S. Carnivora In *Cenozoic Mammals of Africa* (Eds. Werdelin, L. & Sanders, W. J.) 603–657 (University of California Press, Berkeley, 2010).
14. Djaparidze, V. et al. Der altpaleolitishe Fundplatz Dmanisi in Georgien. *Jahr RGZM* **36**, 67–116 (1989).
15. Coil, R. et al. Spatial patterning of the archaeological and paleontological assemblage at Dmanisi, Georgia: An analysis of site formation and carnivore-hominin interaction in Block 2. *J. Hum. Evol.* **143**, 102773 (2020).
16. Gabunia, L. & Vekua, A. A Plio-Pleistocene hominid from Dmanisi, East Georgia, Caucasus. *Nature* **373**, 509-512(1995).
17. Gabunia, L., Vekua, A., Lordkipanidze, D. The environmental contexts of early human occupation of Georgia (Transcaucasia). *J. Hum. Evol.* **38**, 785-802 (2000).
18. Gabunia, L. et al. Earliest Pleistocene hominid cranial remains from Dmanisi, Republic of Georgia: taxonomy, geological setting, and age. *Science* **288**, 1019-1025 (2000).
19. Pillans, B., & Gibbard, P. *The quaternary period. In The geologic time scale 2012*, (Eds. F. M. Gradstein, J. G. Ogg, M. D. Schmitz, G. M. Ogg) 979-1010 (2012).
20. B. Martínez-Navarro, *Revisión sistemática y estudio cuantitativo de la fauna de macromamíferos del yacimiento de Venta Micena (Orce, Granada).* PhD dissertation, Universitat Autònoma de Barcelona, 1991.
